# New Oral Anticoagulants for Thromboprophylaxis after Elective Total Hip and Knee Arthroplasty

**DOI:** 10.1155/2010/280731

**Published:** 2010-07-29

**Authors:** Richard J. Friedman

**Affiliations:** Department of Orthopaedic Surgery, Roper Hospital, Charleston Orthopaedic Associates, 1012 Physicians Drive, Charleston, SC 29414, USA

## Abstract

Anticoagulant drugs reduce the risk of venous thromboembolic events after total hip and knee arthroplasty. However, the use of current drugs, such as low molecular weight heparins, is hampered by their subcutaneous route of administration. The use of vitamin K antagonists is hampered by the requirement for routine coagulation monitoring and dose titration to provide effective anticoagulation without an increased risk of bleeding and numerous food and drug interactions. Clearly, there is a need for new oral, fixed-dose anticoagulant drugs that do not require coagulation monitoring, while demonstrating similar or better efficacy and safety profiles when compared with current agents.

## 1. Introduction

In 2007, the annual number of total hip and knee arthroplasties (THAs and TKAs) in the US was 250,000 and 500,000, respectively [1]. These numbers are expected to increase to 572,000 and 3.48 million for primary THA and TKA, respectively, between 2005 and 2030 [1]. Orthopaedic surgeons and internists are fully aware of these expected increases in the number of elective THAs/TKAs.

The types of patients undergoing THA/TKA are consistent and the risks of surgery are well characterized. Antibiotic prophylaxis for THA/TKA is estimated to decrease the relative risk of wound infection by 81% compared with no prophylaxis [2]. Similarly, the appropriate use of anticoagulant drugs has been shown to reduce the risk of venous thromboembolism (VTE) after THA/TKA, and guidelines recommend their routine use after this type of surgery. Without prophylaxis, the incidence of venographic deep vein thrombosis (DVT) and of pulmonary embolism (PE) after THA are 42–57% and 0.9–28%, respectively [3]. The index event usually occurs at a mean of 21.5 (standard deviation 22.5) days after surgery—typically after hospital discharge [4]. The risk of venographic DVT and PE after TKA is 41–85% and 1.5–10%, respectively [3]. Clinical symptomatic events usually occur at a mean of 9.7 days after TKA and 21.5 days after THA [4], with 75% occurring after a median hospital stay of 5 days for THA. The current trend is towards much shorter hospital stays, with a mean of less than 3 days for THA and TKA at Roper Hospital (Charleston, SC, USA) in 2009, meaning that the vast majority of symptomatic events will occur on an outpatient basis and, therefore, prophylaxis is mainly an outpatient issue.

The American College of Chest Physicians (ACCP) guidelines recommend prophylaxis with anticoagulants for a minimum of 10 days and up to 35 days after THA to reduce the risk of VTE (Grade 1A). After TKA, the ACCP recommends prophylaxis with anticoagulants for at least 10 days (Grade 1A) and suggests up to 35 days in some patients (Grade 2B) [3]. Options include vitamin K antagonists (VKAs), such as warfarin, low molecular weight heparins (LMWHs), such as enoxaparin, and the synthetic pentasaccharide fondaparinux. Although the antiplatelet acetylsalicylic acid (ASA) is considered by some clinicians to have a role in the prevention of PE, its use alone for thromboprophylaxis is not recommended by the ACCP.

The American Academy of Orthopaedic Surgeons (AAOS) has published guidelines strictly on the prevention of PE, not DVT prophylaxis, recommending that patients at standard risk of both PE and major bleeding should be considered for one of the prophylactic agents evaluated in their guideline, including ASA, LMWHs, synthetic pentasaccharides and warfarin. Those at increased (above standard) risk of PE and standard risk of major bleeding should be considered for one of the prophylactic agents evaluated in their guideline, including LMWHs, synthetic pentasaccharides, and warfarin. Patients at standard risk of PE and at increased risk of major bleeding should be considered for prophylaxis with ASA or warfarin, as evaluated in their guideline [5]. However, they fail to provide any definitions or guidelines regarding what patients are at increased risk of bleeding and increased risk of PE, or the standard risk of bleeding and PE.

Although the AAOS does not specifically give guidance on the prevention of DVT after THA/TKA, DVT prophylaxis is as important as the prevention of PE because after an initial DVT (any cause), patients have a 10% risk of recurrent VTE after 1 year [6]. The risk of recurrence is ≤3% per year in patients with transient risk factors (such as recent surgery) [7]. Following an episode of DVT, there is an approximate 24% risk of postthrombotic syndrome after 3 years [6]. Of all untreated initial calf vein thrombi (distal DVT), 20% extend proximally [8]. Moreover, thrombus resolution is slower and postthrombotic syndrome is more severe after proximal than distal DVT [9].

The clinical challenges that orthopaedic surgeons, internists, and clinicians face are that current anticoagulants are administered subcutaneously or require monitoring and dose titration to provide effective anticoagulation without increasing bleeding risk. More effective and convenient alternative anticoagulants, which can be given at fixed doses without routine coagulation monitoring, could improve current clinical practice [10, 11]. New oral anticoagulant drugs are being developed that address these issues, while having similar or better efficacy and safety profiles when compared with current agents.

This paper will review the unmet clinical needs with current agents, discuss the new classes of oral agents, present data on the new oral agents currently available in the European Union (EU) and other countries, and discuss how these agents might meet the needs of orthopaedic surgeons and internists in VTE prophylaxis.

## 2. Current Anticoagulant Regimens

### 2.1. Parenteral Anticoagulants

**Figure 1:**
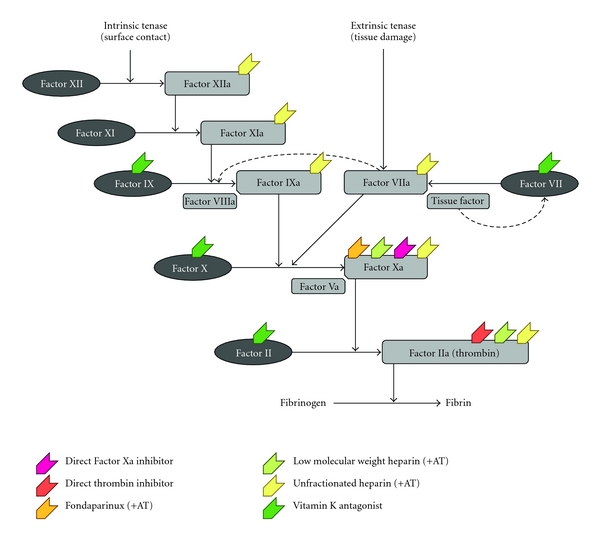
Coagulation cascade: where anticoagulants act. AT, antithrombin.

Although unfractionated heparins (UFHs) have been available since the early 1930s, studies in the 1970s demonstrated that they prevented VTE and fatal PE in patients undergoing surgery [12, 13]. UFHs act at several points of the coagulation cascade (Figure 1). Parenteral LMWHs, which emerged in the early 1980s, also act at several levels of the coagulation cascade [14] (Figure 1).

During the 1990s, a comprehensive series of studies demonstrated the clinical value of LMWHs in reducing the risk of VTE [15] (Table 1). Compared with UFHs, LMWHs offered a convenient solution—they were available as fixed doses, did not require routine coagulation monitoring or dose adjustment [14], and led to clinically significant reductions in the number of venous thromboembolic events [15]. The different LMWHs are created chemically or by depolymerization of UFH. LMWHs target both Factor Xa and Factor IIa (thrombin) [14]. The ratio of Factor Xa: Factor IIa inhibition differs between the different available LMWHs and these ratios are considered to be related to safety and efficacy (the greater the level of Factor Xa inhibition, the greater the efficacy [16] because Factor Xa is generated earlier in the coagulation cascade than thrombin). The ratio of Factor Xa  :  Factor IIa inhibition ranges from 2 : 1 to 4 : 1 for the different LMWHs in current use, compared with 1 : 1 for UFH [14], indicating that antithrombotic activity may be higher when using LMWHs, without the increased risk of bleeding.

Fondaparinux (2.5 mg once daily), a subcutaneously administered, indirect Factor Xa inhibitor (Figure 1), was more effective than enoxaparin (30 mg every 12 hours after TKA and 40 mg once daily after THA) in reducing the risk of VTE [17, 18]. The timing of fondaparinux administration affected the efficacy and incidence of bleeding events after THA/TKA: major bleeding was significantly higher in patients who received their first dose <6 hours after skin closure (3.2%) than in those where the first dose was delayed to ≥6 hours (2.1%, *P* = .045). This effect was more evident in patients who weighed <50 kg, those >75 years of age, and those with moderate renal impairment [19].

It is important to note that bleeding events are always likely after surgery—affecting approximately 2.4% of patients even when no anticoagulants are used [20]—and anticoagulants do not increase bleeding risk when administered correctly with regards to dosage, timing and concomitant use of other agents that affect bleeding (such as nonsteroidal anti-inflammatory drugs). LMWHs offer a good balance, by reducing the number of venous thromboembolic events while maintaining low bleeding rates. However, recent studies have highlighted that only approximately half of patients in the US receive prophylaxis after THA/TKA at the timing, duration and intensity recommended by the ACCP [3, 21]. Worldwide, 59% of surgical patients (undergoing any type of surgery) at risk of VTE receive ACCP-recommended prophylaxis [22]. Furthermore, the duration of prophylaxis is often shorter than the period in which thromboembolic events occur after surgery [4]. Possible reasons for this are that surgeons may not be aware of the substantial postdischarge risk of thromboembolic events, cost, lack of convenience, and need for monitoring.

### 2.2. Oral Antithrombotics

Developed in the 1950s, the VKAs, such as warfarin, indirectly inhibit the production of several coagulation factors (Figure 1). Although recommended in the ACCP guidelines, studies have shown that warfarin is not as effective as parenteral anticoagulants in reducing the venographic DVT incidence [15] (Table 1). Although it is an oral agent, warfarin is less convenient than parenteral anticoagulants, mainly due to the need for frequent monitoring and dose adjustments, and food and drug interactions. Owing to its slow onset of action, it can take 2–4 days for a therapeutic international normalized ratio (INR; range: 2–3 [3]) to be reached. Warfarin has an unpredictable pharmacological profile and dosing needs to be individualized. With a narrow window for safety and efficacy, coagulation monitoring is essential to ensure that patients remain within the INR range after discharge; patients have to be taught how to monitor their INR and take the correct dose at home or frequently attend clinics or a primary care physician. Furthermore, warfarin has many food and drug interactions that may potentiate or inhibit its action, which may be problematic in patients taking concomitant medications for comorbid conditions [23].

A recent study showed that although pharmacy acquisition costs of warfarin are lower than subcutaneous anticoagulant drugs, the total 6-month costs were lower with subcutaneous anticoagulant drugs. Therefore, the initial savings may be offset by a higher incidence of venous thromboembolic events and higher 6-month medical costs with warfarin [24].

The use of ASA remains controversial. It is important to note that ASA is an antiplatelet and not an anticoagulant, but some clinicians consider it to have a role in the prevention of fatal PE and its use is recommended by the AAOS [5] for the prevention of PE only, not for DVT. They recommend that for patients at standard risk of both PE and major bleeding, who represent the majority of patients undergoing total joint arthroplasty, ASA (325 mg twice daily, or 81 mg once daily if gastrointestinal symptoms develop, for 6 weeks) may be one of the prophylactic drugs considered, along with warfarin, LMWH, and fondaparinux. The guidelines do not address other venous thromboembolic events, such as DVT, and do not define standard or increased risk of bleeding or PE. ASA has been shown to reduce venous thromboembolic events by 26% and 13% (relative risk reductions) in patients undergoing THA and TKA, respectively (Table 1) [15], which is less than the reduction with other prophylactic agents.

### 2.3. New Oral Anticoagulants

The ideal anticoagulant needs to be more effective without increasing bleeding risk, safe, convenient to use, administered orally once daily and have fixed dosing—factors that could potentially improve patient compliance. The most promising new oral anticoagulants are the direct thrombin inhibitors (such as dabigatran) and the direct Factor Xa inhibitors (such as rivaroxaban and apixaban)—agents that directly target a single coagulation factor in the coagulation cascade (Figure 1). Dabigatran is approved in the EU and Canada (110 mg within 1–4 hours of surgery, then 220 mg once daily for 28–35 days after THA and 10 days after TKA) for VTE prophylaxis after elective THA/TKA in adults [25]. Rivaroxaban is approved in the EU and numerous other countries for the prevention of VTE in adult patients after elective hip or knee arthroplasty (10 mg 6–10 hours after surgery, then once daily for 35 days after THA and 14 days after TKA [26]). These two drugs represent the first new oral agents for VTE prophylaxis in THA and TKA in over 50 years.

#### 2.3.1. Apixaban

Apixaban is an oral, direct Factor Xa inhibitor with predictable pharmacokinetics and pharmacodynamics [27]. Gender has no clinically relevant effect on apixaban [28]. Data are lacking for the effects of body weight or old age on apixaban. Approximately half of administered apixaban is absorbed and half is recovered in faeces. Of the total dose, approximately one-third is recovered in urine, of which over 80% is apixaban [29]. 

Digoxin and inhibitors or substrates of P450 enzymes do not have clinically relevant interactions with apixaban [30, 31]. Absorption of apixaban is not affected after a high-calorie meal [32]. 

A phase II study [33] of apixaban was used to establish the dose to be used for the phase III clinical development programme. In this study, 1,238 patients were randomized to one of six double-blind apixaban doses (5, 10 or 20 mg once daily or 2.5, 5 or 10 mg twice daily), enoxaparin (30 mg twice daily) or open-label warfarin (titrated to an INR of 1.8–3.0), for 10–14 days. The primary efficacy outcome (the composite of VTE and all-cause mortality) decreased with increasing apixaban dose (*P* = .09 for once-daily or twice-daily regimens combined, *P* = .19 for once-daily and *P* = .13 for twice-daily dosing). There was a significant dose-related increase of total adjudicated bleeding events for the once-daily (*P* = .01) and twice-daily (*P* = .02) regimens. The authors concluded that apixaban 2.5 mg twice daily and 5 mg once daily might have a promising risk–benefit profile compared with enoxaparin 30 mg twice daily and warfarin.

The ADVANCE-1 phase III study compared apixaban 2.5 mg twice daily with the enoxaparin regimen commonly used in North America of 30 mg twice daily, for the prevention of VTE after TKA (*N* = 3,195) [34]. The primary efficacy outcome (composite of venographic DVT, symptomatic, objectively confirmed DVT or nonfatal PE, or death from any cause) occurred in 9.0% of patients receiving apixaban and 8.8% of patients receiving enoxaparin (relative risk 1.02, 95% confidence interval [CI] 0.78 to 1.32, *P* = .06 for noninferiority, statistical criteria not met) during the treatment period. The rates of PE were 1.0% in the apixaban group and 0.4% in the enoxaparin group; two PEs were fatal in the apixaban group and none were fatal in the enoxaparin group. Major or clinically relevant nonmajor bleeding occurred in 2.9% and 4.3% of patients receiving apixaban and enoxaparin, respectively (*P* = .03). Major bleeding occurred in 0.7% and 1.4% of patients receiving apixaban and enoxaparin, respectively (*P* = .053). One patient in the enoxaparin group died from bleeding; none of the apixaban group died from bleeding. In the ADVANCE-2 study, which compared apixaban 2.5 mg twice daily with enoxaparin 40 mg once daily (also in patients undergoing TKA; *N* = 3,057), the hypothesis was that apixaban would be noninferior to enoxaparin based on a prespecified margin for the primary efficacy outcome in which the upper limit of the two-sided 95% CI is <1.25 for relative risk and <5.6% for the absolute risk difference [35]. If both criteria were met, superiority was tested. The primary efficacy endpoint (the same as in ADVANCE-1) occurred in 15.1% of the apixaban group and 24.4% of the enoxaparin group (relative risk 0.62, 95% CI 0.51 to 0.74, *P* < .0001 for noninferiority and superiority; absolute risk difference −9.3%, 95% CI −12.7 to −5.8, *P* < .0001 for noninferiority). Two patients receiving apixaban died from PE and one patient receiving enoxaparin died from bleeding. Major or clinically relevant nonmajor bleeding occurred in 3.5% of the apixaban group and 4.8% of the enoxaparin group (*P* = .09). In summary, the findings of these studies suggest that apixaban is significantly more effective than the 40 mg once-daily enoxaparin regimen at reducing the composite of DVT, PE and death by any cause, with no increased risk of major bleeding. In ADVANCE-1, apixaban did not meet the prespecified statistical criteria for noninferiority of efficacy compared with enoxaparin 30 mg twice daily.

#### 2.3.2. Dabigatran Etexilate

Dabigatran is an oral, once-daily, direct thrombin inhibitor that can be given in a fixed oral dose without dose adjustment for age, body weight or gender [36, 37]. It has a rapid onset of action and provides predictable anticoagulation without the need for routine coagulation monitoring [36]. The main elimination pathway is renal excretion, accounting for more than 80% of the systemically available dose of dabigatran [38]. 

Therapeutic doses of dabigatran are unlikely to interact with drugs that are metabolized by the CYP450 system [38]. It has been shown that food delays the time to peak plasma concentration by 2 hours, but does not have a relevant effect on the extent of dabigatran absorption [39]. 

Dose-ranging studies in patients undergoing THA suggested that the therapeutic window was 12.5–300 mg twice daily (BISTRO I [40]) and in patients undergoing THA and TKA the optimal total daily dose was 100–300 mg (BISTRO II [41]).

Two phase III, randomized trials in patients undergoing TKA have been conducted, one with most of its participating centres in the EU and one in North America, comparing dabigatran with enoxaparin. In the European study (RE-MODEL; *N* = 2,101 [42, 43]), once-daily dabigatran (first dose was half of the subsequent dose; subsequent doses were 150 mg and 220 mg in two tablets) was as effective (noninferior) as once-daily enoxaparin (40 mg) for preventing VTE and all-cause mortality in patients undergoing TKA (40.5% of the dabigatran 150 mg group [absolute risk difference versus enoxaparin 2.8%], 36.4% of the dabigatran 220 mg group [absolute risk difference versus enoxaparin −1.3%] and 37.7% of the enoxaparin group), with similar bleeding rates (compared with enoxaparin: *P* = 1.0 for the dabigatran 150 mg dose and *P* = .082 for the dabigatran 220 mg dose). However, in the RE-MOBILIZE study (*N* = 2,615) [43, 44], which used the usual North American enoxaparin regimen of 30 mg twice daily, dabigatran 150 mg and 220 mg showed inferior efficacy to enoxaparin for the primary outcome of total VTE and death (33.7% of the 150 mg group [absolute risk difference versus enoxaparin 8.4%, *P* = .0009 for noninferiority versus enoxaparin], 31.1% of the dabigatran 220 mg group [absolute risk difference versus enoxaparin 5.8%, *P* = .0234] and 25.3% of the enoxaparin group), although bleeding rates were similar between all three groups (0.6% of the 150 mg group, 0.6% of the 220 mg group and 1.4% of the enoxaparin group). The secondary outcome of major VTE (defined as proximal DVT, PE and VTE-related death) occurred in 3.0% of the dabigatran 150 mg group (risk difference with enoxaparin 0.8%, *P* = .36), 3.4% of the dabigatran 220 mg group (risk difference with enoxaparin 1.2%, *P* = .21) and 2.2% of the enoxaparin group.

The RE-NOVATE study compared once-daily dabigatran 220 mg or 150 mg with once-daily enoxaparin 40 mg after THA (*N* = 3,494). Both doses of dabigatran were noninferior to enoxaparin for the composite of total VTE and death (8.6% of the 150 mg group [absolute risk difference versus enoxaparin 1.9%, *P* < .0001 for noninferiority versus enoxaparin], 6.0% of the 220 mg group [absolute risk difference versus enoxaparin −0.7%, *P* < .0001 for noninferiority versus enoxaparin] and 6.7% of the enoxaparin group). Rates of major bleeding did not differ significantly between the groups (1.3% for the 150 mg group [*P* = .60], 2.0% for the 220 mg group [*P* = .44] and 1.6% for the enoxaparin group). There were no significant differences in cardiac events or liver enzyme elevations in any of the three groups [43, 45]. Whereas RE-MODEL and RE-NOVATE showed the tested doses of dabigatran were noninferior to the 40-mg enoxaparin regimen for VTE prophylaxis, RE-MOBILIZE found dabigatran to be inferior to the 30-mg twice-daily enoxaparin regimen. Possible reasons for this finding are the higher daily dosage of enoxaparin and longer treatment duration in the RE-MOBILIZE study compared with the RE-MODEL study.

A meta-analysis of the three dabigatran studies (which assessed the recommended 220 mg dose) supported the findings of RE-MODEL and RE-NOVATE [46]. It showed that there were no significant differences between dabigatran 220 mg and enoxaparin in any endpoints when RE-MODEL and RE-NOVATE were analysed (*P* > .15), or when all three trials were included in the analysis (all *P* > .30). Risk ratios (random effects) for the composite of total VTE and all-cause mortality were 0.95 (95% CI 0.82 to 1.10) in the two-trial analysis and 1.05 (95% CI 0.87 to 1.26) in the three-trial analysis. Major bleeding rates did not differ significantly when RE-MODEL and RE-NOVATE were analysed (*P* = .41 for random-effects and fixed-effects analyses) or when all three studies were analysed (*P* = .85 for random-effects and *P* = .95 for fixed-effects analyses). 

In a recent prespecified pooled analysis of the studies, the primary outcome (composite outcome of major and VTE-related mortality) occurred in 3.3% of the enoxaparin group, 3.8% of the 150 mg group (risk difference 0.5%, 95% CI −0.6 to 1.6, I^2^ = 0% [if I^2^ was greater than 50%, heterogeneity was considered to be substantial]) and 3.0% of the dabigatran 220 mg group (risk difference versus enoxaparin −0.2%, 95% CI −1.3 to 0.9, I^2^ = 37%). Rates of major bleeding were 1.4% in the enoxaparin group, 1.1% in the 150 mg group (risk difference −0.4%, 95% CI −1.0 to 0.2, I^2^ = 0%) and 1.4% in the dabigatran 220 mg group (risk difference −0.2%, 95% CI −0.8 to 0.5, I^2^ = 40%). These findings suggest that dabigatran was as effective as enoxaparin and the risk of major bleeding was similar [47].

#### 2.3.3. Rivaroxaban

Rivaroxaban—an oral, direct Factor Xa inhibitor—was found to exhibit a predictable pharmacokinetic and pharmacodynamic profile and does not require dose adjustment for age, gender [48] or weight [49]. Rivaroxaban and its metabolites have a dual route of elimination: one-third of the administered drug is cleared as unchanged active drug by the kidneys; one-third is metabolized to inactive metabolites and then excreted by the kidneys; and one-third is metabolized to inactive metabolites and then excreted by the faecal route [50].

Rivaroxaban has a low propensity for drug–drug interactions with frequently used concomitant medications, such as naproxen [51], ASA [52] or clopidogrel [53], and no interaction with the cardiac glycoside digoxin [54]. Dietary restrictions are not necessary and rivaroxaban was given with or without food in the phase III VTE prevention studies (RECORD1–4 [REgulation of Coagulation in ORthopaedic surgery to prevent Deep vein thrombosis and pulmonary embolism]).

Phase II studies [55–58] showed that all investigated rivaroxaban dose regimens had similar efficacy to enoxaparin, and the incidence of major bleeding was not significantly different to enoxaparin across a fourfold dose range (5–20 mg total daily rivaroxaban dose).

The RECORD programme comprised four phase III studies investigating the efficacy and safety of rivaroxaban in 12,500 patients undergoing THA and TKA [59–62]. All patients received rivaroxaban 10 mg once daily 6–8 hours after surgery, and there was no upper age or weight limit for participation. The primary efficacy endpoint was the composite of DVT, nonfatal PE and all-cause mortality up to day 30–42 after surgery for RECORD1 and RECORD2, up to day 13–17 for RECORD3 and up to day 17 for RECORD4. The main safety endpoint was the incidence of treatment-emergent (observed no later than 2 days after the last dose of the study drug) major bleeding events. Other safety outcomes (for example, nonmajor bleeding and postoperative wound infection) were also reported [59–62].

RECORD1 showed that 5 weeks of extended-duration rivaroxaban (10 mg once daily for 31–39 days after surgery) was significantly more effective than enoxaparin (40 mg once daily for 31–39 days) for extended-duration prophylaxis in patients undergoing THA (1.1% versus 3.7% for the primary efficacy endpoint, *P* < .001) [59]. Major bleeding events did not differ significantly between the groups (0.3% versus 0.1% of patients, *P* = .18). Clinically relevant nonmajor bleeding occurred in 2.9% of the rivaroxaban group versus 2.4% of the enoxaparin group; haemorrhagic wound complications in 1.5% versus 1.7% of patients; and postoperative wound infections in 0.4% of patients in both groups. The incidence of symptomatic VTE during treatment was not significantly different between the groups (0.3% versus 0.5%, *P* = .22).

RECORD2 demonstrated that extended-duration rivaroxaban prophylaxis (10 mg once daily for 31–39 days after surgery) was significantly more effective than short-duration prophylaxis with enoxaparin (40 mg once daily for 10–14 days) followed by placebo in patients undergoing THA (2.0% versus 9.3% for the primary efficacy endpoint, *P* < .0001) [60]. The incidence of bleeding was comparable between extended-regimen rivaroxaban and short-duration enoxaparin. Major bleeding events occurred in <0.1% of patients in both groups. Clinically relevant nonmajor bleeding was recorded in 3.3% of the rivaroxaban group versus 2.7% of the enoxaparin group; haemorrhagic wound complications in 1.6% versus 1.7% of patients; and postoperative wound infections in 0.7% versus 0.5% of patients, respectively. Significantly fewer patients in the rivaroxaban group had symptomatic VTE (0.2%) than in the enoxaparin group (1.2%, *P* = .004) during the active study period.

In RECORD3, rivaroxaban prophylaxis (10 mg once daily for 10–14 days) was significantly more effective than the European enoxaparin regimen for prophylaxis (40 mg once daily) in patients undergoing TKA (9.6% versus 18.9% for the primary efficacy endpoint, *P* < .001), with a similar safety profile [61]. Rates of major bleeding were similar in the rivaroxaban and enoxaparin groups (0.6% versus 0.5%, *P* = .77); clinically relevant nonmajor bleeding occurred in 2.7% versus 2.3% of patients; haemorrhagic wound complications in 2.0% versus 1.9% of patients; and postoperative wound infections in 0.6% versus 0.9% of patients. There was a significant reduction in the number of symptomatic venous thromboembolic events in the rivaroxaban group (0.7% versus 2.0%, *P* = .005).

In RECORD4, rivaroxaban showed significantly better efficacy than the enoxaparin regimen (30 mg every 12 hours) commonly used in North America for short-term prophylaxis after TKA (6.9% versus 10.1%, respectively, for the primary efficacy endpoint, *P* = .0118) [62]. The rates of major bleeding were 0.7% versus 0.3%  (*P* = .1096), respectively; clinically relevant nonmajor bleeding occurred in 2.6% versus 2.0% of patients; haemorrhagic wound complications in 1.4% versus 1.5% of patients; and postoperative wound infections in 0.3% versus 0.2% of patients, respectively. The observed incidences of symptomatic VTE in those receiving rivaroxaban or enoxaparin were 0.7% versus 1.2%  (*P* = .187), respectively.

In the four studies comparing rivaroxaban with enoxaparin, rivaroxaban demonstrated superior efficacy compared with enoxaparin. In addition, extended thromboprophylaxis with rivaroxaban was significantly more effective than short-term enoxaparin plus placebo in the prevention of total, major and symptomatic VTE after THA. Furthermore, the incidence of treatment-emergent major and clinically relevant nonmajor bleeding was low for rivaroxaban and enoxaparin (*P* = .21 [data on file] for RECORD1, *P* = .39 [data on file] for RECORD2, *P* = .44 for RECORD3 [61] and *P* = .18 for RECORD4 [62]). There was no evidence of compromised liver function or rebound cardiovascular events associated with rivaroxaban.

In a pooled analysis of the RECORD1, 2 and 3 studies (which compared rivaroxaban with enoxaparin 40 mg once daily after THA and TKA) [63], the prespecified primary efficacy outcome (the composite of symptomatic VTE [DVT or PE] and all-cause mortality at 2 weeks) was 0.4% and 0.8%, respectively (*P* = .005). The rates were 0.5% and 1.3%, respectively, at the end of the planned medication period (*P* < .001). Rates of on-treatment major bleeding were 0.2% for both drugs at 2 weeks (*P* = .662), and 0.3% for rivaroxaban and 0.2% for enoxaparin at the end of the planned medication period (*P* = .305). Rates of clinically relevant nonmajor bleeding were 2.6% for rivaroxaban and 2.3% for enoxaparin at 2 weeks, and 3.0% and 2.5%, respectively, at the end of the planned medication period (*P*-values not reported).

In a pooled analysis of all four RECORD studies [64], the primary efficacy endpoint (the composite of symptomatic VTE [DVT or PE] and death) was significantly reduced for the rivaroxaban regimens compared with enoxaparin regimens at day 12 ± 2 (0.5% versus 1.0%, *P* = .001), in the planned treatment period (0.6% versus 1.3%, *P* < .001), and in a post hoc analysis of the treatment and follow-up period (0.8% versus 1.6%, *P* < .001). Rates of treatment-emergent major bleeding were not significantly different between groups at any of the time points analysed [64]. The composite of major and clinically relevant nonmajor bleeding did not differ at day 12 ± 2 (*P* = .186), but was significantly higher for rivaroxaban in the planned medication period (*P* = .039). Rates of the composite of PE and death were lower for rivaroxaban compared with enoxaparin in the planned treatment period and follow-up (0.5% versus 0.8%, *P* = .039) [65].

Future research needs to assess whether changing the timing of the first dose could improve the safety profile without significantly affecting efficacy. In theory, the earlier an anticoagulant is given, the better the efficacy, but at a cost of increased bleeding [66]. Conversely, the longer anticoagulation is delayed, the lower the risk of bleeding, but efficacy may decrease too.

## 3. Summary and Conclusions

Among the numerous oral anticoagulants currently in phase II and III development, three of the oral agents—apixaban, dabigatran and rivaroxaban—hold considerable potential benefits for improving thromboprophylaxis strategies. In light of recent promising findings, more studies on direct thrombin inhibitors and Factor Xa inhibitors are likely. In addition, reports from daily clinical practice will indicate whether the new agents will change current practice [67]. A phase III TKA study has shown that apixaban is significantly more effective than the once-daily enoxaparin regimen, without an increase in bleeding. The phase III studies comparing dabigatran with enoxaparin were designed to show the noninferiority of dabigatran. It was found that dabigatran has similar efficacy and safety compared with the once-daily enoxaparin regimen in THA and TKA. In addition, phase III studies have shown significantly improved efficacy and similar safety for rivaroxaban compared with both once-daily and twice-daily enoxaparin regimens in THA and TKA. All of these agents provide the benefit of oral dosing without the need for monitoring or dose adjustment, thereby improving the convenience of prophylaxis.

## Figures and Tables

**Table 1:** Deep vein thrombosis frequencies following prophylaxis with different agents after total hip and knee arthroplasties [15].

	DVT frequency after total hip arthroplasty		DVT frequency after total knee arthroplasty	
	Prevalence (%)	Relative risk reduction (%)	Prevalence (%)	Relative risk reduction (%)
Acetylsalicylic acid	40.2	26	56.0	13
Low molecular weight heparins	16.1	70	30.6	52
Warfarin	22.1	59	46.8	27

DVT, deep vein thrombosis.
